# The roles of thermal insulation and heat storage in the energy performance of the wall materials: a simulation study

**DOI:** 10.1038/srep24181

**Published:** 2016-04-07

**Authors:** Linshuang Long, Hong Ye

**Affiliations:** 1 Department of Thermal Science and Energy Engineering, University of Science and Technology of China, Hefei, Anhui 230027, PR China

## Abstract

A high-performance envelope is the prerequisite and foundation to a zero energy building. The thermal conductivity and volumetric heat capacity of a wall are two thermophysical properties that strongly influence the energy performance. Although many case studies have been performed, the results failed to give a big picture of the roles of these properties in the energy performance of an active building. In this work, a traversal study on the energy performance of a standard room with all potential wall materials was performed for the first time. It was revealed that both heat storage materials and insulation materials are suitable for external walls. However, the importances of those materials are distinct in different situations: the heat storage plays a primary role when the thermal conductivity of the material is relatively high, but the effect of the thermal insulation is dominant when the conductivity is relatively low. Regarding internal walls, they are less significant to the energy performance than the external ones, and they need exclusively the heat storage materials with a high thermal conductivity. These requirements for materials are consistent under various climate conditions. This study may provide a roadmap for the material scientists interested in developing high-performance wall materials.

The total final energy consumption worldwide increased from 4,672 Mtoe (million tons of oil equivalent, 1 Mtoe = 4.1868 × 10^4^ trillion joule) to 8,979 Mtoe between 1973 and 2012. China was responsible for 7.9% of the world’s total consumption in 1973, and this proportion increased to 19.1% in 2012 (data from *2014 Key World Energy Statistics* published by International Energy Agency). In 2011, the energy consumption ratio of buildings to that of the country as a whole was 19.74% in China^1^. The application of Zero Energy Buildings (ZEBs) has been perceived as a promising way to reduce both energy consumption and carbon dioxide emission^2^,^3^,^4^,^5^. Basically, a ZEB is an advanced building whose operating energy is negligible or can be offset by the renewable energy generation while providing a satisfying thermal comfort degree. Despite the accurate definition of ZEB is still ambiguous^6^, a high-performing building envelope is the prerequisite and foundation to a ZEB^7^.

A building envelope includes, generally, two parts: the transparent and opaque ones. The transparent parts of the building envelope are usually optimized on the aspect of their radiation properties^8^ and thermal insulation performance^9^. The non-transparent parts of the envelope can be further divided into two types: the external ones in direct contact with the outdoor environment (including solar radiation, outdoor air, etc.) and the internal ones. Widely investigated strategies to optimize the opaque envelope are to enhance their heat storage capacity as well as thermal insulation performance^10^,^11^,^12^.

Cabeza *et al*., for example, conducted a set of experiments to approve the performance of high internal thermal inertia in Mediterranean climates^13^,^14^, and Stazi *et al*. emphasized the efficiency of external insulations in the same climate^15^,^16^,^17^,^18^. Most of these investigations were carried out experimentally or numerically with the method of case studies, which are effective to give a direct comparison among particular cases. However, the meaning of the results is inevitably restrained: only a few types of materials or configurations of walls can be compared and evaluated. With these limited results, the effects of thermal insulation and heat storage on energy performance can hardly be investigated comprehensively and there is always a lack of a big picture of the overall scene.

Different from the above mentioned case studies, Zhang and his group have provided an inverse problem method^11^,^19^,^20^,^21^,^22^ and an optimization method based on the concept of entransy^23^ to determine the ideal thermophysical properties of the walls. Nevertheless, these methods, accompanied by a lot of mathematical derivations, are relatively complicated, and a general relationship between the energy consumption and various wall materials cannot be established through them.

Due to the fact that the thermal insulation and heat storage are inherent and concomitant capacities of the envelopes, some questions remain to be addressed. How do these capacities of a wall affect the energy performance of a building? Does there exist any interaction between these capacities? How do the results diversify for external and internal walls? To answer these questions, one requires an overall concept about the roles of the thermal insulation and heat storage in the energy performance of the envelopes.

Painting such a big picture of wall materials implies that a thorough investigation on buildings is needed. However, a building consists of a massive number of configurations, including the size, orientation, type of window, window-to-wall ratio, internal loads, schedule, etc., making a thorough study that contains all configurations inaccessible. On the other hand, any walls within different configurations can be grouped into external ones and internal ones according to whether they are directly exposed to the outdoor environment. As a result, despite the varied configurations of buildings, a room with both external and internal walls is a rational and typical physical representation of a building to investigate the overall effect of wall materials. Windows and internal heat gains of the standard room were excluded at first to focus on the opaque part of building envelopes as well as to further simplify the model. Although the study of such a special room is meaningful for the opaque envelopes, the effect of windows and heat gains are still included later to examine the universality of the study. Climate conditions may also influence the results of the investigation, so three types of climates (the climate of hot summer and cold winter, that of cold climate and that of hot summer and warm winter) are considered.

In this work, we aim to provide a traversal study on the energy performance of the standard room with all potential wall materials for the first time. However, how can we label varieties of materials and then find out all potential materials? From the aspect of engineering thermophysics, a wall material can be characterized by the thermophysical properties such as thermal conductivity, *k*, mass density, *ρ* and specific heat capacity, *c*_*p*_, and these parameters are naturally of interest. As already known, *k* measures the ability of a material to conduct thermal energy. The other two parameters, *ρ* and *c*_*p*_, are always multiplied together in the energy balance equations (seen in the supplementary information), so they can be integrated into a single parameter: volumetric heat capacity, *C*_V_, which measures the heat storage capacity of a material per unit volume. To facilitate a comprehensive investigation that includes all potential wall materials, *k* from 0.001 to 5 W/(m·K) and *C*_V_ from 50 to 5000 kJ/(m^3^·K) were considered, resulting in 2160 types of materials with diverse combinations of *k* and *C*_V_. The energy performances of the opaque envelopes made of each material were calculated with a simulation tool named BuildingEnergy.

## Results

### Materials for external walls

All potential materials of *k* and *C*_V_ within the aforementioned ranges were computed in BuildingEnergy as the external or internal walls. The room was assumed to locate in Hefei, China, where the cooling/summer season is from June 15th to September 5th and the heating/winter season is from December 5th to March 5th of the following year. The climate data used in BuildingEnergy were the typical yearly meteorological data provided by the Chinese Architecture-specific Meteorological Data Sets for Thermal Environment Analysis. The thicknesses of the external and internal walls were set as 240 and 100 mm, respectively, and other wall thickness can be equivalently converted into these values through a treatment described in the supplementary information. Owing to such a treatment, the conclusions from the fixed thicknesses will be universal for all values of thicknesses.

**Figure 1:**
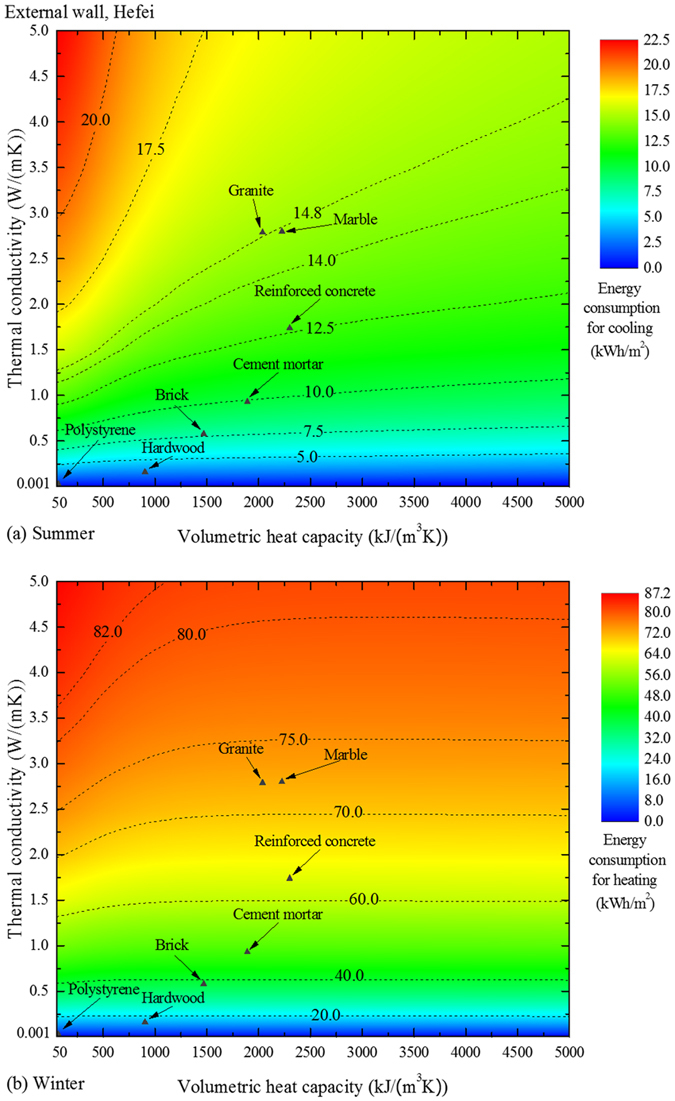
Energy consumption contours related to the external walls. When the materials of the external wall vary by their thermal conductivity and volumetric heat capacity, the internal-wall materials stay unchanged. (**a**) The results for summer in Hefei and (**b**) those for winter in Hefei. Several common building materials are also located in the figures according to their properties.

Figure 1 shows the energy consumption contours for the external walls made of different materials, in which the materials of the internal walls are fixed as common bricks. The thermophysical properties of the brick are provided in Table 1. As Fig. 1 depicts, both the thermal conductivity and volumetric heat capacity of the external-wall materials exert a significant impact on the energy performance, and the energy consumption varies extensively along with *k* and *C*_V_. A value of zero can be achieved for an extremely low *k*, due to the absence of a window and internal heat source.

For the summer application (Fig. 1(a)), generally, either a decrease in conductivity or an increase in volumetric heat capacity of the materials causes a reduction in cooling energy consumption of the room. A low *k* and a high *C*_V_ imply a small thermal diffusivity *α*, which is defined as *k*/*C*_V_ or *k*/(*ρc*_*p*_). *α* affects the transient thermal conduction process through a wall: in materials of a small α, heat transfers sluggishly, and thus the outdoor environment has a smaller influence on the indoor environment than the situation with materials of a great α. In addition to retarding the thermal conduction within the wall through a small *α*, a low *k* also contributes to blocking the heat transfer across the boundary of the external wall. If *k* is low enough, the heat may rarely reach the indoor-side surface from the outdoor environment, so *C*_V_ cannot exert its effect on the heat transfer process across the interior. As a consequence, when *k* is lower than 0.25 W/(m·K) in Fig. 1(a), the contour lines are nearly horizontal, implying that *C*_V_ has a negligible effect on the energy performance and that a low *k* takes priority over a great *C*_V_.

As *k* increases, the slopes of the contour lines also increase, namely, the significance of *C*_V_ is increasing. When *k* is higher than 3.0 W/(m·K), the lines are nearly vertical, which means that the energy performance is almost exclusively affected by *C*_V_. Such a phenomenon may be explicated from the lumped capacitance approximation. When this approximation holds, i.e., the assumption of a uniform temperature distribution within the solid is reasonable, the temperature gradients within the solid may be neglected, therefore the change in thermal conductivity has an insignificant influence on the heat conduction. Basically, lumped capacitance approximation is satisfied for a situation that the resistance to conduction within the solid is much less than the resistance to convection between the surface and the fluid^24^. In our case, if *k* is high enough, the wall may behave as a lumped capacitance solid, making the energy performance influenced individually by *C*_V_.

For the winter application (Fig. 1(b)), the general tendency of how the material properties affect the energy performance is consistent with that in summer, but the slopes of the contour lines are almost zero when *C*_V _ ≳ 2000 kJ/(m^3^·K), indicating that *C*_V_ has a limited influence in winter.

Some typical building materials, whose properties are presented in Table 1, are also plotted in Fig. 1. When made of one of these materials, the corresponding external wall is diverse in energy performance. The trend is, ordinarily, that the energy consumption reduces with the decreasing conductivity. For close values of *k* (the granite and marble, for example), the energy consumption is determined by *C*_V_: the material with a higher *C*_V_ leads to a lower energy consumption.

**Figure 2:**
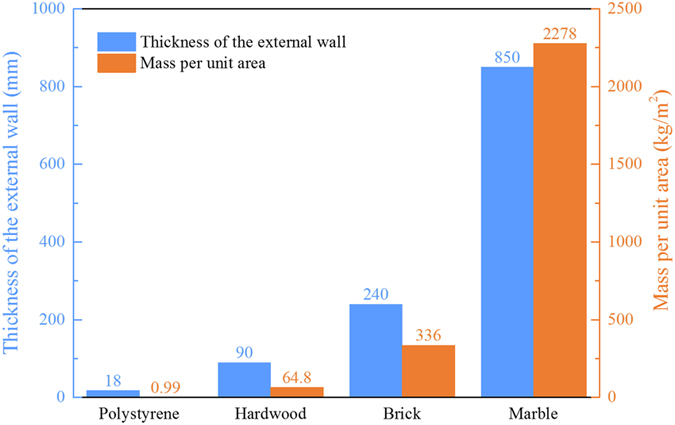
Comparisons in thickness and mass per unit wall area of typical materials. The energy performances of an external wall made of different materials are close to that of 240 mm-thickness bricks. For example, the cooling energy consumption of a room with an 850 mm-thickness-marble external wall is approximately equal to that with a 240 mm-thickness-brick external wall.

As mentioned above, the energy performance in Fig. 1 was discussed with a fixed thickness of the walls. In practical situations, the thickness with the same energy performance may also be a reference parameter. Figure 2 illustrates the comparisons of thickness and mass for some typical materials, whose cooling energy performances approximate that of a 240 mm brick wall. The thickness of the polystyrene is just 2% of the marble and 7.5% of the brick. Furthermore, the mass per unit wall area of the polystyrene wall is much smaller than those of the other materials due to a low density of the polystyrene. A small mass per unit area means a lower construction cost, and a smaller thickness results in larger net area. Therefore, an external wall made of light insulation materials, like polystyrene, will be recommendable in buildings following an improvement in mechanical strength.

### Materials for internal walls

**Figure 3:**
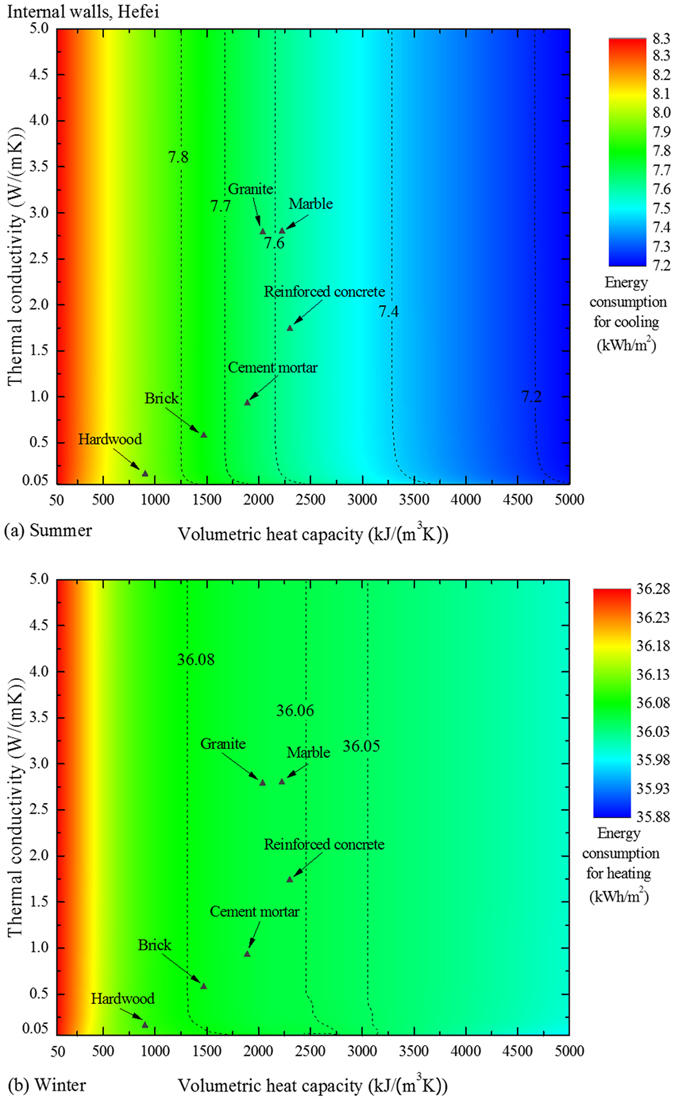
Energy consumption contours related to the internal walls. When the materials of the internal walls vary, the external-wall materials stay unchanged. (**a**) The results for summer in Hefei and (**b**) those for winter in Hefei. Several common building materials are also positioned in the figures.

Now we consider the energy performance of the internal-wall materials. Similar contour map are presented in Fig. 3, in which the external-wall materials are common bricks. It can be observed that the energy consumption decreases as *k* increases when *k* ≲ 0.5 W/(m·K). A high *k* facilitates the thermal conduction. In summer, for example, the temperature of the indoor-side surface can be lowered through the conduction of some heat into the interior of the wall, resulting in a reduction in energy consumption for cooling (as Eq. (8) in the supplementary information explicates). For materials of *k* higher than 0.5 W/(m·K), the contour lines are vertical so the energy performance is influenced exclusively by the volumetric heat capacity. The increase in *C*_V_ causes the reduction in both cooling and heating energy consumptions. Regarding the materials in Table 1, the reinforced concrete, whose volumetric heat capacity is the highest, is the best candidate for the internal-wall material.

Note that when *k* and *C*_V_ vary, the energy consumption varies from 7.2 to 8.3 kWh/m^2^ in summer, and the range is 35.88 ~ 36.28 kWh/m^2^ in winter. Nevertheless, the corresponding ranges in Fig. 1 are 0 ~ 22.5 and 0 ~ 87.2 kWh/m^2^. The much broader ranges imply a more significant role of the external wall in the energy performance, meanwhile, a greater potential for improvement.

Thermal conductivity and volumetric heat capacity are inherent thermophysical properties of a material. Nonetheless, materials are embodied in some building components, such as a wall, a window, a floor, etc. For this reason, engineers prefer employing the parameters which are able to describe a whole component to particular materials. Overall heat transfer coefficient, also termed *U*-value, and total heat capacity are customarily used to characterize the thermal insulation performance and heat storage capacity of a wall, respectively. With the analysis elaborated in the supplementary information, the requirements for the wall materials may also be stated as the demand for the wall as a whole component, which can be summarized as: the total heat capacities of both the external and internal walls should be high, and the *U*-value of the external wall should be low.

### Effects of windows and internal heat gains

**Figure 4:**
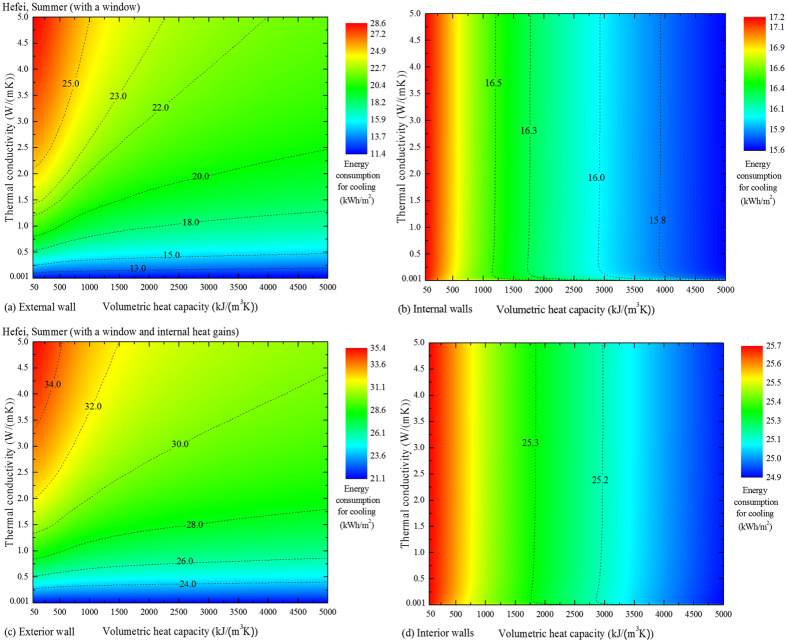
Cooling energy consumption of various materials for a room with a window and internal heat gains in Hefei. (**a,b**) The room has a single-glazed window with a size of 1.5 m×1.5 m. (**c,d**) In addition to the window, internal heat gains are also considered. These figures may generalize the discoveries for more practical situations.

As previously declared, we have ignored the potential influence of the window until now. Here Fig. 4(a,b) depict the performances of a room having a window. The single-glazed window, which locates in the center of the external wall, has a size of 1.5 × 1.5 m^2^ and a solar transmittance of 77%. Comparing the situations with and without a window, it is discovered that the presence of the window raises the cooling energy consumption, but does not change the trend of how the wall materials influence the energy performance. Due to the absence of a window, the lowest energy consumption that can be obtained through the improvement in the external wall is zero in Fig. 1(a), while the corresponding value with a window is 11.4 kWh/m^2^ in Fig. 4(a). The gap between the lower limits is generated by the transparent part of the envelope, i.e., the window, and may be filled by the continuous development of the windows, revealing that a high-performance building envelope should be achieved by the simultaneous improvements in the transparent and opaque parts.

To further generalize the results, internal heat gains were also considered in the room with a window to simulate a more realistic situation. The heat gain from the occupants and equipment is taken to be 4.3 W per unit of floor area, and that from the lighting is 3.5 W per unit of floor area while the lights are on, from 18:00 until 22:00 each day. The results are plotted in Fig. 4(c,d), which illustrate that the consideration of internal heat gains does not change the general rules of the influence of wall materials on the energy performance. The effects of other configurations of the room on the general rules, e.g., the orientation, the room size, were also proved to be negligible, and the details can be seen in the supplementary information.

### Effects of climate conditions

**Figure 5:**
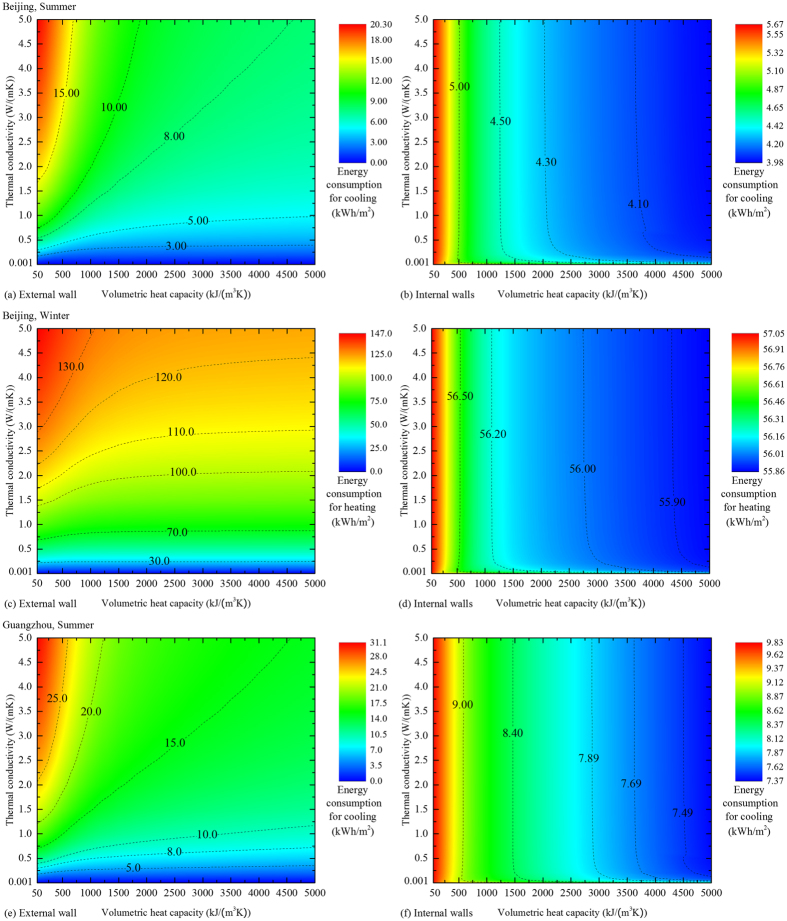
Effect on energy consumption of external- and internal-wall materials in different climate regions. (**a,d**) The results for Beijing with a cold climate and (**e,f**) those for Guangzhou with a climate of hot summer and warm winter. The unchanged rules of properties under different climates extrapolate the results.

The foregoing discussions were established for the city of Hefei, which has a climate of hot summer and cold winter. To examine the effect of climates, Fig. 5 shows the situations for Beijing with a cold climate, and Guangzhou with a climate of hot summer and warm winter. A heating period is absent for Guangzhou due to the fact that the average temperature of the coldest month is still 14 °C. The trends of the influence of the material properties on the energy consumption are utterly the same as those in Hefei, which means that these trends are independent from climates. The only difference occurs in the energy consumption ranges: rooms in Guangzhou exhibit a higher cooling consumption than those in Hefei, and rooms in Beijing have a higher heating consumption. The results for more extreme climates are presented in the supplementary information, and the general trends are still consistent.

## Discussion

In this study, the effects of thermal conductivity and volumetric heat capacity of the wall materials on the energy performance were investigated, which elucidated the roles of thermal insulation and heat storage of the external and internal walls in an active building through a traversal study and theoretical analysis.

An energy-saving internal wall needs a large heat storage capacity, as well as a high *k* which helps the heat storage/release process. However, an internal wall has a less significant influence on the energy performance than the external one does. For an external wall, in most cases, both the thermal insulation and heat storage can strongly affect the energy performance—materials of a low thermal conductivity and a high volumetric heat capacity, i.e., a small thermal diffusivity, are favorable to energy efficiency in buildings. When the thermal conductivity of the material is 3.0 W/(m·K) or higher, the heat storage plays a primary role, but its influence vanishes when *k* is lower than 0.3 W/(m·K). *k* is anticipated to be as low as possible, and its importance is more prominent in winter than in summer. In addition, the requirements for the wall materials are universal and independent from climates and the inclusion of transparent building envelopes.

With these theoretical guidelines, suggestions on the improvement of the actual wall materials can be given now. An effective external wall requires materials with excellent thermal insulation and large heat storage capacity. However, the role of the heat storage seems to have been underestimated before, but the discoveries from this study reveal that heat storage materials, e.g., phase change materials^25^,^26^, are also suitable for the external walls. Insulation materials, which are well-known to function as the external-wall materials, perform well due to the fact that they obstruct the heat transfers both across the boundary and within the medium of the wall, and those with high mechanical strength, e.g., NanoCon^27^ (a new material with a nano pore structure, which has both a low thermal conductivity and a construction property as good as concrete), will be an ideal choice for the future external walls. Regarding internal walls, materials with large heat storage capacity are also demanded, and heat storage materials have already been widely applied to internal walls. It should be noted that the thermal conductivity of the materials need to be enhanced to make full use of their heat capacity.

## Methods

### Description of the room

The standard room is assumed in a middle story of a multi-story apartment building. The room has internal dimensions of 4 × 4 × 4 m^3^ and contains one single external wall facing towards the south. The other walls, the ceiling and the floor are not directly exposed to the outdoor environment. The thickness of the external wall is 240 mm and that of the interior ones is 100 mm. The indoor temperature of the room is maintained with heating, ventilation and air conditioning (HVAC) facilities at 18 and 26 °C in the heating and cooling seasons, respectively, as per the industry standard of China, JGJ 134–2010 (*Design standard for energy efficiency of residential buildings in hot summer and cold winter zone*).

### Ranges of thermal conductivity and volumetric heat capacity

The lower limit of the thermal conductivity of building components is reportedly achieved by a vacuum insulation panel (VIP). With a porous core wrapped with a multilayer envelope, a VIP is one of the most high-performance insulation components, whose effective thermal conductivity can be as low as 0.002 W/(m·K)^28^,^29^,^30^. In practical applications, the thermal conductivity of some rocks is high in relation to other building materials except metals and can be set as the upper limit. For example, a quartzite (Sioux) has a conductivity of 5.38 W/(m·K) (adapted from Appendix A^24^). In this study, we set the thermal conductivity in the range from 0.001 to 5 W/(m·K).

The mass density of building materials is usually lower than 3000 kg/m^3^, and the specific heat capacity is usually less than 3000 J/kg (except phase change materials during their melting process). However, the materials with a high density generally have a low specific heat capacity and those with high specific heat capacity often have a low density. For instance, a marble (Halston) has a high density of 2680 kg/m^3^, while a specific heat capacity of 830 J/kg; a yellow pine wood has a high specific heat capacity of 2805 J/kg, whereas a density of 640 kg/m^3^ (adapted from Appendix A^24^). These facts make the product of the density and specific heat capacity, i.e., the volumetric heat capacity, approximately lower than 3000 kJ/(m^3^·K). Conservatively, the upper limit of the volumetric heat capacity is set as 5000 kJ/(m^3^·K). However, this upper limit is still much lower than the volumetric heat capacity of phase change materials, which generally have a much higher heat storage capacity than the sensible thermal storage materials. For a PCM, such as paraffin, its equivalent volumetric heat capacity during the phase transition process can be as high as 8 × 10^4^ kJ/(kg·K). However, instead of being investigated in this study, application performance of PCMs will be amid our future research. The lower limit of *C*_V_ is set as 50 kJ/(m^3^·K), referring to a polyurethane. Namely, the range of the volumetric heat capacity is from 50 to 5000 kJ/(m^3^·K).

### BuildingEnergy program

The energy performance of the room is simulated using an energy modeling program called BuildingEnergy. This program is compiled with a non-steady-state heat transfer model, in which the building envelope and the indoor air and outdoor air are divided into hundreds of nodes. For each node, the energy conservation equation is based on the implicit difference method. The equations for all of the nodes in the temperature field form a matrix. The temperature field is determined by solving the matrix via the Gauss–Seidel iteration method. The physical and numerical models employed in the program are elaborated in the supplementary information of this study.

BuildingEnergy has been validated using ANSI/ASHRAE Standard 140-2004 (“Standard Method of Test for the Evaluation of Building Energy Analysis Computer Programs”) in our previous work^31^. We also validated the program through a series of experiments conducted in full-size rooms^32^,^33^, and the details of the validation are displayed in the supplementary information.

## Additional Information

**How to cite this article**: Long, L. and Ye, H. The roles of thermal insulation and heat storage in the energy performance of the wall materials: a simulation study. *Sci. Rep.* **6**, 24181; doi: 10.1038/srep24181 (2016).

## Supplementary Material

Supplementary Information

## Figures and Tables

**Table 1:** Thermophysical properties of typical building materials.

Materials	Thermal conductivity [W/m·K]	Volumetric heat capacity [kJ/m^3^·K]	Specific heat capacity [J/kg·K]	Mass density [kg/m^3^]
Polystyrene^a^	0.027	66.55	1210	55
Hardwood^a^	0.16	903.6	1255	720
Brick, common^b^	0.58	1470	1050	1400
Cement mortar^b^	0.93	1890	1050	1800
Reinforced concrete^b^	1.74	2300	920	2500
Granite, Barre^a^	2.79	2038.25	775	2630
Marble, Halston^a^	2.80	2224.4	830	2680

^a^Adapted from Appendix A^24^.

^b^Adapted from Appendix 4 of GB 50176 (Thermal Design Code for Civil Building, National standards of People’s Republic of China).
