# Supplementary material for: The roles of thermal insulation and heat storage in the energy performance of the wall materials: a simulation study
Source: Sci Rep. 2016 Apr 7;6:24181. doi: 10.1038/srep24181 (PMC4823728; doi:10.1038/srep24181)
Supplement: Supplementary Information [file srep24181-s1.pdf]

Supplementary Information

The roles of thermal insulation and heat storage in the energy performance of the wall materials: a simulation study

Linshuang Long, Hong Ye*

Department of Thermal Science and Energy Engineering, University of Science and Technology of China, Hefei, Anhui 230027, PR China

*Corresponding author: hye@ustc.edu.cn, Tel. /Fax: +86 551 63607281

This document provides some detailed information for a more comprehensive understanding of our study. It includes *Physical and numerical models*, *Experimental validation of BuildingEnergy*, *Treatment of the thickness of the walls* and *Perspective of engineers*.

Nomenclature

A	area of the surface [m^2]	Greek letters	
ACH	Air changes per hour	α	thermal diffusivity [m^2/s]
C	total heat capacity [J/K]	δ	thickness [m or mm]
C_V	volumetric heat capacity [J/(m^3·K) or kJ/(m^3·K)]	ρ	mass density [kg/m^3]
c_p	specific heat capacity at constant pressure [J/kg]	Subscripts	
$h_{i\text{-air}}$	convection heat transfer coefficient between surface i and air [W/(m^2·K)]	air,i	indoor air
k	thermal conductivity [W/m·K]	air,o	outdoor air
q''	heat flux [W/m^2]	cond	thermal conduction
q	instantaneous load for space cooling or heating [W]	conv	thermal convection
$R_{i\text{-}j}$	thermal resistance for radiation between surface i and j [K/W]	e	external wall
r_{vent}	ventilation rate [ACH]	e,i	ith node of the external wall
S'	solar irradiation absorbed by the wall per unit area [W/m^2]	i	internal wall
T	temperature [K or °C]	i,i	ith node of the internal wall
Δt	time interval [s]	j	surface in a room
U	overall heat transfer [W/K]	m	surface of outdoor surroundings (e.g., the sky, the ground)
V	volume [m^3]	rad	thermal radiation
Δx	spatial interval [m]		

1. Physical and numerical models

Here we will elaborate the heat transfer processes and corresponding models for the walls. Two primary simulation assumptions are made in BuildingEnergy: (1) the heat transfer across the building envelope is one-dimensional; and (2) the indoor temperature of the room and that of the adjacent rooms is the same, so the center surfaces of the interior walls are adiabatic.

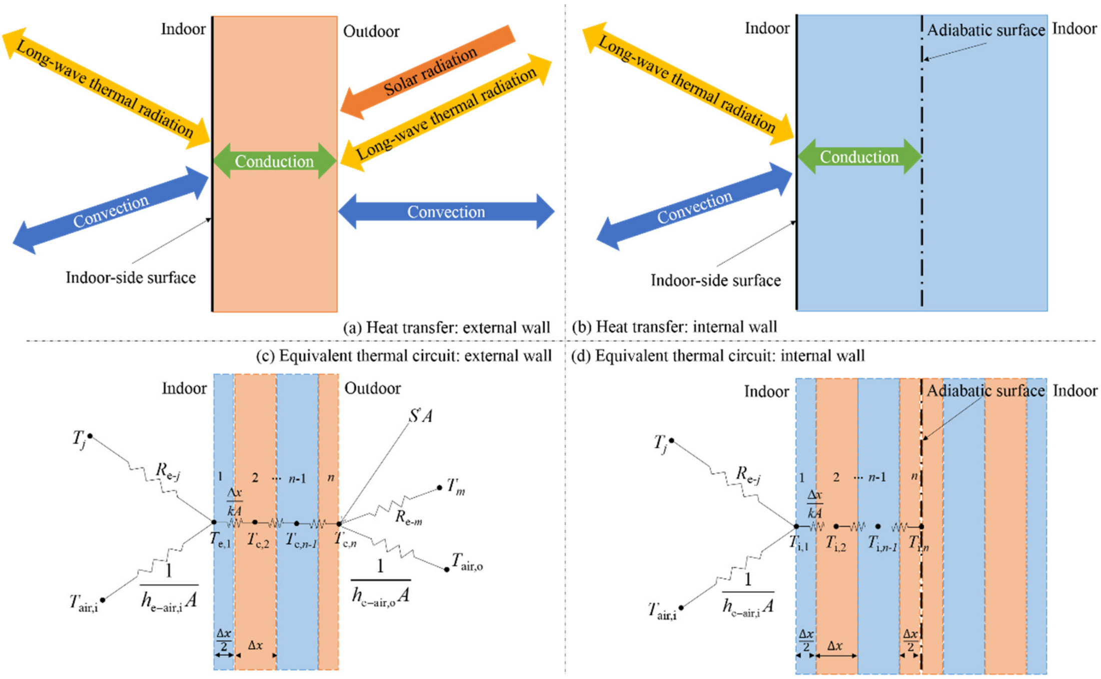

Figure S 1 Schematic diagrams of the heat transfer processes and equivalent thermal circuits for the external (a, c) and internal (b, d) walls.

As Figure S 1 (a) and (b) depict, on the indoor side, both the external and internal walls transfer energy with the indoor air by convection and with other surfaces in the room by the long-wave thermal radiation. On the outdoor side, the external wall receives the solar radiation, transfers energy with the outdoor air by convection and with the outdoor surroundings by the long-wave thermal radiation. In the center of the internal walls, there exists an adiabatic surface because of the foregoing assumption of the equal indoor temperature in the adjacent rooms. Across the walls, energy is transferred by conduction. Divided into nodes, the walls are now discretized in space, as shown in Figure S 1 (c) and (d). For the node at the indoor surface of the external wall, i.e., Node 1 in Figure S 1 (c), the energy balance equation can be expressed as

$$\rho c_p A \frac{\Delta x}{2} \frac{T_{\mathrm{e},1}^{p+1} - T_{\mathrm{e},1}^{p}}{\Delta t} = kA \frac{T_{\mathrm{e},2}^{p} - T_{\mathrm{e},1}^{p}}{\Delta x} + \sum_{j} \frac{T_j^{p} - T_{\mathrm{e},1}^{p}}{R_{\mathrm{e}-j}} + h_{\mathrm{e-air,i}} A \left(T_{\mathrm{air,i}}^{p} - T_{\mathrm{e},1}^{p} \right) \tag{1}$$

where the superscript p is used to denote the time dependence of T, and the time derivative is expressed in terms of the difference in temperatures associated with the *new* (p+1) and *previous* (p) times. From the energy balance perspective, the left-hand side of Equation (1) represents the rate of increase of thermal energy stored in the node-controlled volume. The

terms on the right-hand side are, in sequence, the heat flows entering the node from the adjoining node by conduction, that from the other surfaces in the room by the long-wave thermal radiation, and that from the indoor air by convection.

For the node at the outdoor surface of the external wall, i.e., Node n in Figure S 1 (c), the energy balance equation is expressed as

$$\rho c_p A \frac{\Delta x}{2} \frac{T_{\mathrm{e},n}^{p+1} - T_{\mathrm{e},n}^{p}}{\Delta t} = kA \frac{T_{\mathrm{e},n-1}^{p} - T_{\mathrm{e},n}^{p}}{\Delta x} + S^{'} A + \sum_{m} \frac{T_{m}^{p} - T_{\mathrm{e},n}^{p}}{R_{\mathrm{e}-m}} + h_{\mathrm{e-air,o}} A \left(T_{\mathrm{air,o}}^{p} - T_{\mathrm{e},n}^{p} \right) \quad (2)$$

where the four terms on the right-hand side are the heat flows entering the node from the adjoining node by conduction, that from the sun by absorption, that from the outdoor surrounding by the long-wave thermal radiation, and that from the outdoor air by convection.

For the node at the indoor surface of the internal wall, i.e., Node 1 in Figure S 1 (d), the energy balance equation is written as

$$\rho c_p A \frac{\Delta x}{2} \frac{T_{\mathrm{i},1}^{P+1} - T_{\mathrm{i},1}^{P}}{\Delta t} = kA \frac{T_{\mathrm{i},2}^{P} - T_{\mathrm{i},1}^{P}}{\Delta x} + \sum_{j} \frac{T_{j}^{P} - T_{\mathrm{i},1}^{P}}{R_{\mathrm{i}-j}} + h_{\mathrm{i-air,i}} A \left(T_{\mathrm{air,i}} - T_{\mathrm{i},1}^{P} \right) \quad (3)$$

The energy balance equation for the node on the adiabatic surface of the internal wall, i.e., Node n in Figure S 1 (d), is expressed as

$$\rho c_p A \frac{\Delta x}{2} \frac{T_{\mathrm{i},n}^{P+1} - T_{\mathrm{i},n}^{P}}{\Delta t} = kA \frac{T_{\mathrm{i},n-1}^{P} - T_{\mathrm{i},n}^{P}}{\Delta x} \quad (4)$$

For the internal nodes of both the external and internal walls, the energy balance equations can be written in a single discrete form as

$$\frac{T_i^{p+1} - T_i^{p}}{\Delta t} = \frac{k}{\rho c_p} \frac{T_{i-1}^{p} + T_{i+1}^{p} - 2T_i^{p}}{\Delta x^2} \qquad 2 \le i \le n-1 \quad (5)$$

which is also known as the discrete heat diffusion equation. The subscript i denotes the ith node of the external or internal wall.

In these equations, k, ρ and c_p are relevant to the thermophysical properties of the wall materials and are the parameters of interest. As already known, k measures the ability of a material to conduct thermal energy. It can be observed that ρ and c_p are always multiplied together, so they can be grouped into a single parameter: volumetric heat capacity C_V, which measures the heat storage capacity of a material per unit volume.

2. Experimental validation of BuildingEnergy

The program was validated through a series of experiments in a testing platform located in the campus of University of Science and Technology of China, which is illustrated in Figure S 2 (a). The platform contains two identical testing rooms with internal dimensions of 2.9×1.8×1.8 m^3 (length ×width ×height), one of which is shown in Figure S 2 (b). The non-transparent envelopes of the rooms are made of polyurethane wrapped with metal boards, in which the polyurethane is 37 kg/m^3 in density, 1385 J/(kg·K) in specific heat and 0.0228 W/(m·K) in thermal conductivity. The thicknesses of the walls and the roofs are 10 cm.

The experiment began on July 7th, 2012 in Hefei, China and lasted for 7 days. The indoor temperature of the room was maintained at 20 °C through the input of cool/heat wind from the fan coil units. The load needed to maintain the indoor temperature, meteorological data (e.g., solar irradiance, wind speed, ambient temperature, etc.) during the experiment were

measured and recorded. With the meteorological data and the thermophysical properties of room envelopes, the load was then simulated via BuildingEnergy. The simulated and measured loads are compared in Figure S 2 (c), showing an acceptable accuracy of BuildingEnergy program.

Another experiment was conducted from October 2nd to October 6th, 2013. Different with the aforementioned one, this verification focused on the accuracy of the simulation of indoor temperature. The simulated and measured results are plotted in Figure S 2 (d), in which the simulation occurred to be practically identical to measured data.

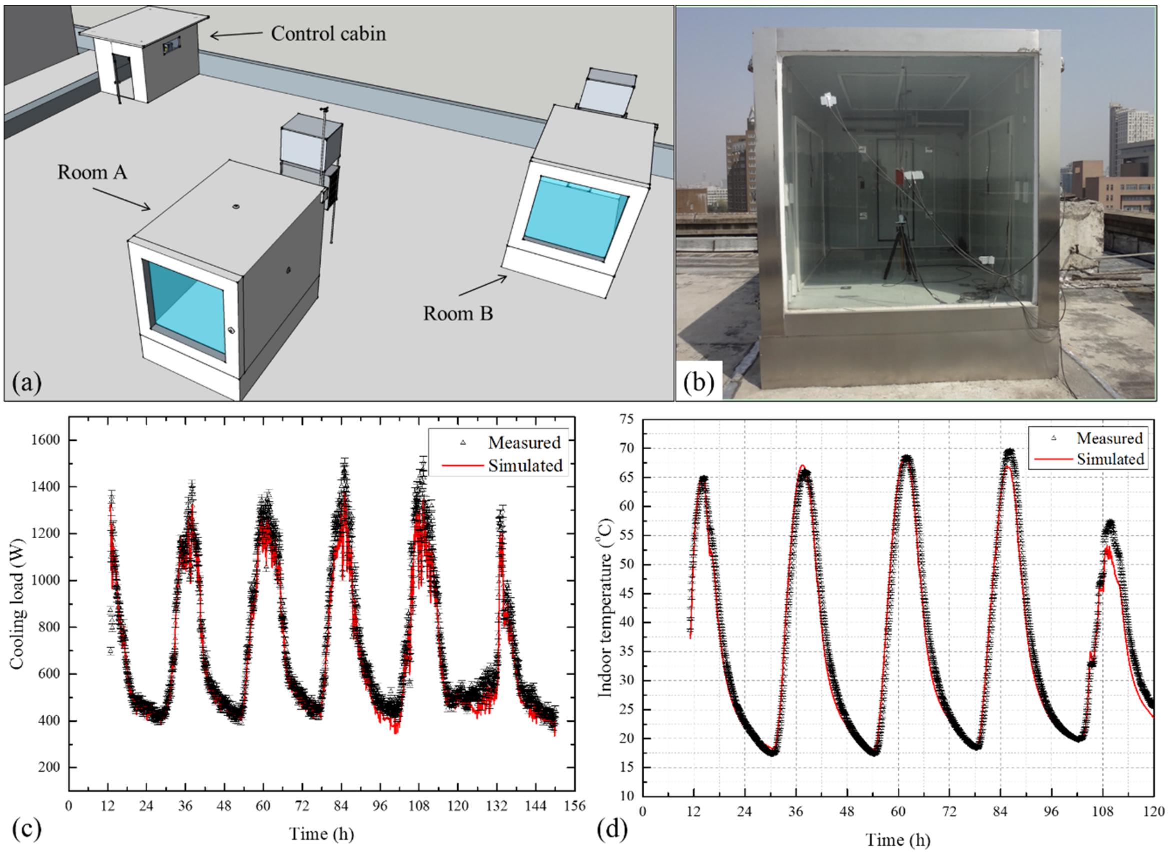

Figure S 2 Validation of BuildingEnergy program.

3. Treatment of the thickness of the walls

In addition to the thermal conductivity and volumetric heat capacity of the material that constitutes a wall, the thickness, δ, also plays an important role in the performance of the walls. However, it is complicated to analyze the performance of walls that identified by three variables. To simplify the analysis by reducing a variable, we will employ a treatment of δ, in which the influence of δ is incorporated into that of k and C_V. With the help of this treatment, we can hold the thickness of the walls constant and discuss the effects of k and C_V on the performance, and the results may also be adapted to any other thicknesses.

In Equation (1)~(5), δ is embodied through the spatial interval Δx and the number of nodes n under a relationship of $\delta = \Delta x \times n$. Here we consider two walls with thicknesses of δ_1 and δ_2. If both of them are divided into n nodes, their spatial intervals should be Δx_1 and $\Delta x_2 = (\delta_2/\delta_1)\cdot\Delta x_1$. For the wall with δ_2, Equation (1)~(5) can be expressed as

$$C_V A \frac{(\delta_2/\delta_1)\Delta x_1}{2} \frac{T_j^{P+1} - T_j^P}{\Delta t} = kA \frac{T_m^P - T_j^P}{(\delta_2/\delta_1)\Delta x_1} + \cdots \quad \begin{cases} \text{if } j = 1 \text{ then } m = 2 \\ \text{if } j = n \text{ then } m = n-1 \end{cases} \tag{6a}$$

$$\frac{T_i^{p+1} - T_i^p}{\Delta t} = \frac{k}{C_V} \frac{T_{i-1}^p + T_{i+1}^p - 2T_i^p}{\left[(\delta_2/\delta_1)\Delta x_1\right]^2} \tag{6b}$$

where Equation (1)~(5) for each type of nodes have been classified into two types of equations: that for the surface nodes (6a) and that for the internal nodes (6b). Another simplification has been made by absenting the terms irrelevant to the thickness. Equation (6) can also be rearranged as

$$\left[(\delta_2/\delta_1) C_V\right] A \frac{\Delta x_1}{2} \frac{T_j^{P+1} - T_j^P}{\Delta t} = \left[(\delta_1/\delta_2) k\right] A \frac{T_m^P - T_j^P}{\Delta x_1} + \cdots \tag{7a}$$

$$\frac{T_i^{p+1} - T_i^p}{\Delta t} = \frac{\left[(\delta_1/\delta_2) k\right]}{\left[(\delta_2/\delta_1) C_V\right]} \frac{T_{i-1}^p + T_{i+1}^p - 2T_i^p}{\Delta x_1^2} \tag{7b}$$

Due to the absolute equivalence between Equation (6) and (7), the temperature filed determined from these equations are the same, indicating that each node depicted in Figure S 1 (c) or (d) of one wall is at the same temperature with the corresponding node of the other wall, namely, $T_{1,i} = T_{2,i}$ (i = 1, …, n) where the subscript 1 or 2 denotes the wall controlled by Equation (6) or (7). Specifically, the wall with a thickness of $\Delta x_1 \times n$ or δ_1 and properties of $(\delta_1/\delta_2) \cdot k$ and $(\delta_2/\delta_1) \cdot C_V$, which subjected to Equation (7) and labeled as *Wall-1*, has an identical temperature distribution of the nodes with that of $(\delta_2/\delta_1) \cdot \Delta x_1 \times n$ or δ_2, k and C_V, which related to Equation (6) and gained a tag of *Wall-2*. The temperature distribution in turn determines the energy performance of a wall through

$$q = \sum_j h_{j-\text{air,i}} A_j \left(T_{\text{air,i}} - T_j\right) + r_{\text{vent}} \rho_{\text{air,i}} c_{\text{p,air,i}} V_{\text{air,i}} \left(T_{\text{air,o}} - T_{\text{air,i}}\right) \tag{8}$$

where q is the instantaneous load for space cooling or heating, and T_j is the temperature of each surface in a room. The room that contains *Wall-1* and the cooling or heating load that needed in the room are separately designated as Room-1 and q_1 to facilitate the presentation. Likewise, the denotations of *Room-2* and q_2 are associated with *Wall-2*. In Equation (8), the surface temperature T_j, i.e., temperature of the first node in Figure S 1 (c) or (d), of *Wall-1* are equal to that of *Wall-2* because of their equivalent temperature distributions. Due to the fact that the only difference between *Room-1* and *-2* lies in *Wall-1* and *-2*, each T_j in *Room-1* including the surface temperature of *Wall-1* is the same with the corresponding one in *Room-2* containing that of *Wall-2*, revealing the equivalence between q_1 and q_2. In other words, the wall with a thickness of δ_2 and a material of k and C_V has the same energy performance as the wall with a thickness of δ_1 and a material of $(\delta_2/\delta_1) \cdot k$ and $(\delta_1/\delta_2) \cdot C_V$, meaning that a variation in δ may be incorporated into a change in k and C_V. With this useful treatment, the thicknesses of the walls can be normalized into a same value to reduce a variable. From another aspect, results for a single thickness may also be converted into those for any other thicknesses.

4. Perspective of engineers

Thermal conductivity and volumetric heat capacity are inherent thermophysical properties of a material. Nonetheless, materials are objectified as some building components, such as a wall, a window, a floor, etc. For this reason, engineers prefer employing the parameters describing a whole component to particular materials. Overall heat transfer coefficient, also termed *U*-value, and total heat capacity are customarily used to characterize the thermal insulation performance and heat

storage capacity of a wall, respectively. The total heat capacity per unit wall area is obtained from

$$C = \delta \cdot C_V = \delta \cdot \rho \cdot c_p \tag{9}$$

U-value per unit area is defined as

$$U = \frac{1}{1/h_{\text{e-air,i}} + \delta/k + 1/h_{\text{e-air,o}}} \tag{10}$$

From Equation (10), it is evident that U-value is related to the total thermal resistance for the heat transfer between the outdoor air and the indoor air by conduction and convection. As convection is beyond the scope of this study, U-value depends entirely on the thickness and thermal conductivity. Equation (10) also implies that the U-value is invalid for an internal wall because no heat transfer occurs across the adiabatic center.

Referring back to the treatment of thickness, the wall with a thickness of δ_1 and a material of k_1 and C_{V1} was equivalent to the wall with a thickness of δ_2 and a material of $(\delta_2/\delta_1)\cdot k_1$ and $(\delta_1/\delta_2)\cdot C_{V1}$. The corresponding U-values and total heat capacities follow that

$$\begin{cases} U_2 = \dfrac{1}{1/h_{\text{e-air,i}} + \delta_2/k_2 + 1/h_{\text{e-air,o}}} = \dfrac{1}{1/h_{\text{e-air,i}} + \delta_2/\left[\left(\delta_2/\delta_1\right)k_1\right] + 1/h_{\text{e-air,o}}} \\ \quad = \dfrac{1}{1/h_{\text{e-air,i}} + \delta_1/k_1 + 1/h_{\text{e-air,o}}} = U_1 \\ C_2 = \delta_2 \cdot C_{V2} = \delta_2 \cdot \left[\left(\delta_1/\delta_2\right) \cdot C_{V1}\right] = \delta_1 \cdot C_{V1} = C_1 \end{cases} \tag{11}$$

The parameters of each wall are equivalent, implying that the principle of the thickness substitute is to maintain the U-value and total heat capacity constant. The requirements for the wall materials may also be stated as the demand for the wall as a whole component. They can be summarized as: the total heat capacities of both the external and internal walls should be high, and the U-value of the external wall should be low.

5. General applicability of the results

The effects of the configurations of the room on the results are demonstrated in this section to evaluate the general applicability of the results through a rigorous way. Several configurations of the room were considered, and their impacts are shown in Figure S 3. In this figure, the effects of the thermophysical properties of the external wall on the energy performance of a room, where the materials of the internal walls were fixed as common bricks and the configurations of each subfigure will be introduced later.

All of the subfigures in Figure S 3 are derived from the basic room discussed in the main text, which contains a window and internal heat gains and is located in Hefei.

In Figure S 3 (a), the external wall faces towards the east, while the basic room's external wall towards the south. In Figure S 3 (b), the volume, size and aspect ratio of the rooms are different from those of the basic room. This room has a dimension of 3 m (height) ×4 m (width) ×5 m (depth), and the corresponding dimension of the basic room is 4 m ×4 m ×5 m. In Figure S 3 (c), the roof of the room is also exposed to the external environment, more than one external wall of the basic room. This room can be also perceived as a room with two external walls. Figure S 3 (d) displays the effect of the window-to-wall ratio. The window of the room in Figure S 3 (d) has a size of 2 m × 2 m, or a window-to-wall ratio of 0.25, while those of the basic room are 1.5 m × 1.5 m and 0.14, respectively. The wall whose properties are discussed in Figure S 3 is one external envelope of the corresponding room, i.e., the east one in (a), the south wall in (b) and (d) and the roof in (c).

Figure S 3 displays only a single type of each configuration, and we have simulated other two or three types of each configurations to further verify the generality of the results. It can be summarized from the work that the general trends of how the properties of the walls influence the energy performance are independent from the configurations of the rooms. In other words, the orientation, number of external walls, volume, sizes and exposed roof have little effect on the results because these configurations of the room do not change the form of the energy balance equations of the walls.

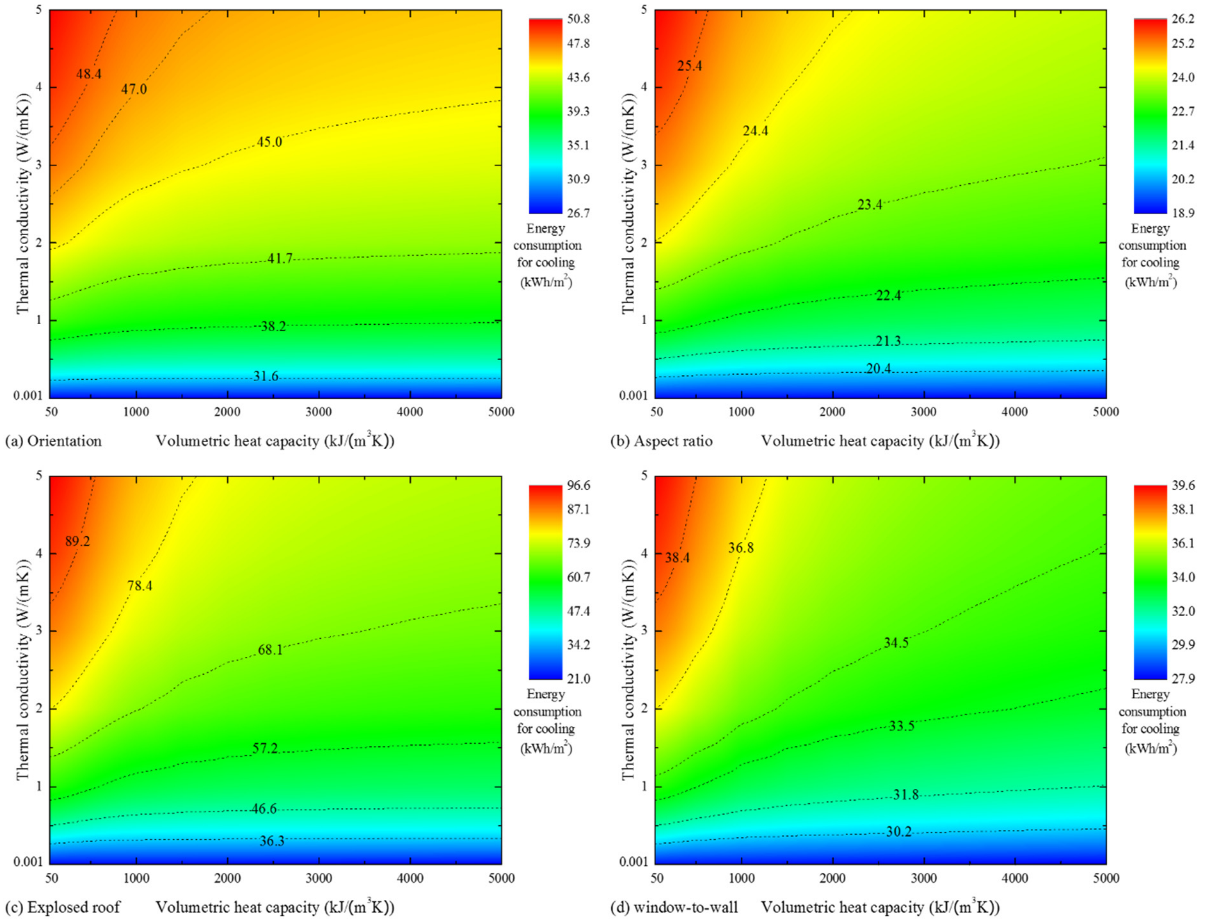

Figure S 3 Results of the rooms with different configurations. Each subfigure presents one type of configurations based on the basic room, which has a dimension of 4 m ×4 m ×4 m and whose exclusive external wall facing towards the south contains a window of 1.5 m × 1.5 m. The external wall room in (a) faces towards the east. The room in (b) has a dimension of 3 m (height) ×4 m (width) ×5 m (depth). The room in (c) contains two external envelopes, the south wall and the roof. The window of the room in (d) has a size of 2 m × 2 m.

6. Results under extreme climates

In addition to the cities of Hefei, Beijing and Guangzhou in the main text, two more Chinese cities, Turpan and Mohe, were chosen to represent the cities under the extreme weather.

Turpan locates 42.93° N, 89.2° E, and is of the desert climate. It shares the record of the highest air temperature that once appeared in China. We considered the hottest three months in Turpan, i.e., June, July and August, during which the variations of the dry-bulb temperature are shown in Figure S 4 (a). The corresponding results are displayed in Figure S 4 (b).

The city of Mohe locates 52.97° N, 122.5° E, and is of the severe cold climate. It is the northernmost city in

China. The variations of the dry-bulb temperature of the coldest three months in Mohe, i.e., January, February and December, are shown in Figure S 4 (c). The results are displayed in Figure S 4 (d). It can be observed from Figure S 4 (b) and (d) that the general trends are still consistent with those in the cities of Hefei, Beijing and Guangzhou in the main text.

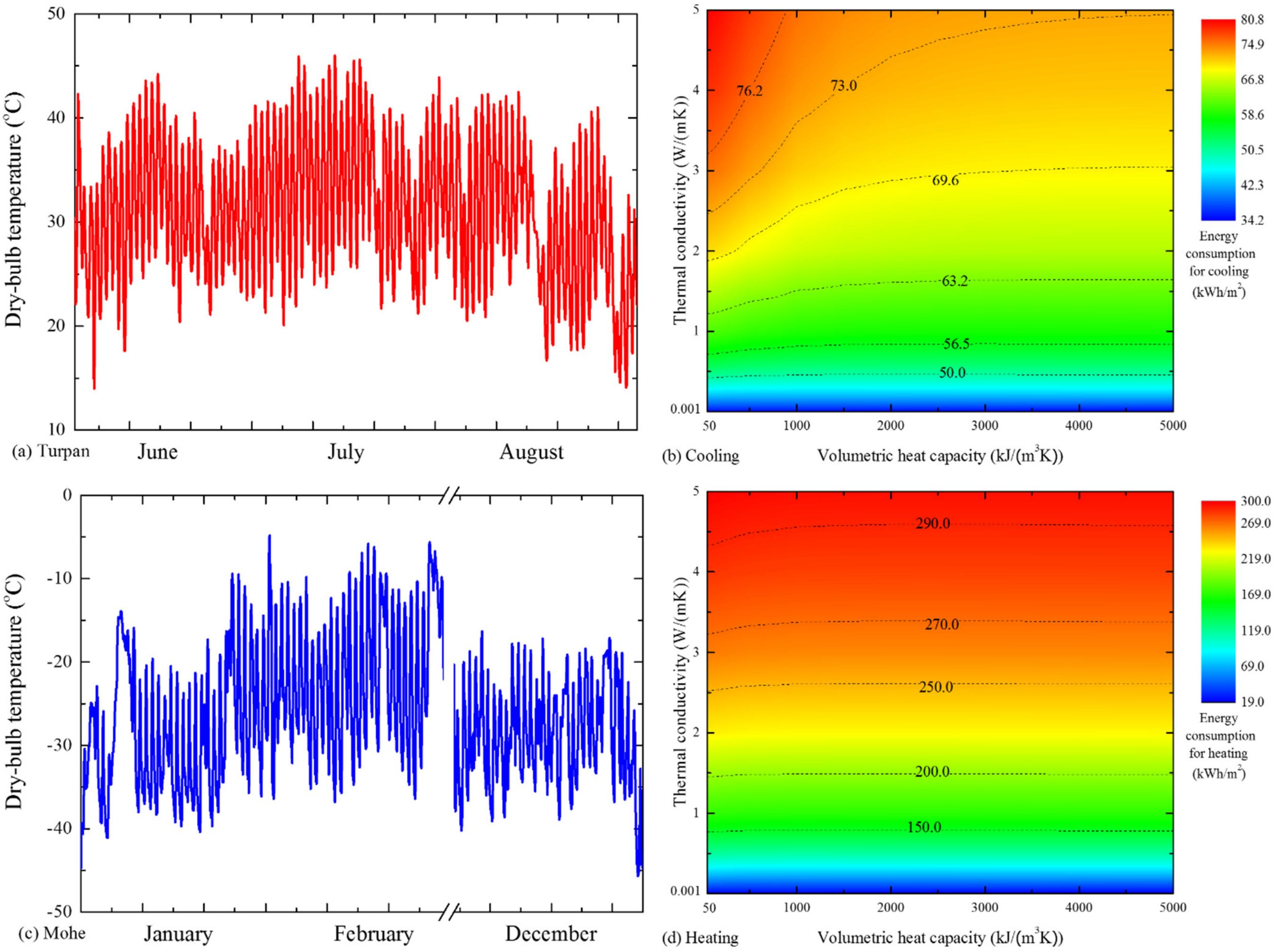

Figure S 4 Results under the extreme climates. The cities of Turpan and Mohe are perceived as typical cities under the extreme hot and extreme cold weathers, respectively. Dry-bulb temperatures in (a) Turpan and (c) Mohe are displayed. The properties of the external wall are discussed, and those of the internal walls are constant. Energy performances in (b) Turpan are considered for summer applications, while those in (d) Mohe for winter applications.
